# Suitable housekeeping genes for normalization of transcript abundance analysis by real-time RT-PCR in cultured bovine granulosa cells during hypoxia and differential cell plating density

**DOI:** 10.1186/1477-7827-12-118

**Published:** 2014-11-27

**Authors:** Vijay S Baddela, Anja Baufeld, Vengala R Yenuganti, Jens Vanselow, Dheer Singh

**Affiliations:** Animal Biochemistry Division, National Dairy Research Institute, Karnal, 132001 Haryana India; Reproductive Biology, Leibniz Institute for Farm Animal Biology (FBN), Wilhelm-Stahl-Allee 2, 18196 Dummerstorf, Germany

**Keywords:** *TBP*, *B2M*, *GAPDH*, *HMBS*, *RPS18*, *RPLP0*, *HPRT1*, External standards, Co-amplification

## Abstract

**Background:**

Bovine granulosa cell culture models are important to understand molecular mechanisms of ovarian function. Folliculogenesis and luteinization are associated with increasing density of cells and local hypoxic conditions. The current study identified two reliable housekeeping genes useful for gene normalization in granulosa cells under different *in vitro* conditions.

**Methods:**

During the current experiments cells were subjected to different biological and physical stimuli, follicle stimulating hormone, different initial cell plating density and hypoxia. Transcript abundance of seven housekeeping genes was quantified by real-time RT-PCR with co-amplification of the respective external standard.

**Results:**

Three of the genes, *GAPDH*, *HMBS*, and *HPRT*1 were found to be regulated by initial cell plating density, five of them, *GAPDH*, *HMBS*, *HPRT*1, *RPLP0* and *RPS18* under hypoxic conditions, but none of them after FSH stimulation. In detail, *GAPDH* was up regulated, but *HPRT1* and *HMBS* were down regulated at high density and under hypoxia. Expression of *RPLP0* and *RPS18* was inconsistent, but was significantly down-regulated in particular at high cell density combined with hypoxia. In contrast, *TBP* and *B2M* genes were neither regulated under different plating density conditions nor by hypoxia as they showed similar expression levels under all conditions analyzed.

**Conclusions:**

The present data indicate that *TBP* and *B2M* are appropriate housekeeping genes for normalization of transcript abundance measured by real-time RT-PCR in granulosa cells subjected to different plating densities, oxygen concentrations and FSH stimulation.

## Background

Ovarian follicles can be considered as the basic units of female reproduction. Each ovarian follicle is composed of spherical aggregations of somatic cells (granulosa and theca cells) and a single oocyte. These follicles are periodically initiated to grow, develop and to culminate in the ovulation of usually a single competent oocyte in monovulatory animals like human and bovine [1]. Ovulation is a complex process which is perfectly regulated by endocrine and paracrine acting factors like steroids, gonadotropins and other proteohormones [2]. As the estrus or menstrual cycle progresses towards ovulation, the circulatory levels of gonadotropins, follicle stimulating hormone (FSH) and luteinizing hormone (LH) increase which further promotes ovulation. We have already shown earlier that granulosa cell (GC) proliferation is increased by FSH and insulin-like growth factor 1 (IGF-I) [3]. As the follicle approaches ovulation, the density of GC increases, but after the preovulatory LH surge, proliferation ceases and GC undergo luteinization thus transforming them from estrogen into progesterone secreting cells [4]. Another important factor associated with the periovulatory perdiod is a change of local oxygen partial pressure. Ovulatory follicles have been shown to undergo hypoxic conditions during the pre-ovulatory stage which is essential for ovulation [5]. Cell density and hypoxia based cell culture experiments are not only important in terms of understanding ovarian physiology during ovulation and luteinization, but may also help to understand cancer cell physiology. Cancer cells rapidly divide and outgrow angiogenic regions of the tumor thus giving rise to regions with significantly lower oxygen partial pressure than found in healthy tissues [6].

It is well established that hypoxia can change gene expression profiles in cultured GC and other cells [7, 8]. But also cell density can remarkably change gene expression and cell physiology [9]. In a recent report, we have demonstrated that initial plating density largely effects gene expression, proliferation and hormone production in cultured bovine GC [10].

Gene expression analysis is an important prerequisite to understand cell physiology and metabolism. However, to minimize methodological errors, which might occur during the process of RNA quantification and cDNA synthesis, it is necessary to select reliable housekeeping genes that are not regulated under the different experimental conditions for normalization [11]. Housekeeping genes are constitutively expressed genes whose expression is required to perform basic cellular biological functions for survival and maintaining integrity of the cell [12]. In spite of the fact that glyceraldehyde-3-phosphate dehydrogenase (GAPDH) and β-actin (ACTB) were routinely used for the normalization of transcript abundance data in real-time RT-PCR experiments, conflicting observations have been reported by various scientific groups regarding their regulation. Both genes have been reported to be regulated differentially in different tissues [13] and even in the same tissue under different physiological conditions [14]. In contrast, in cultured rat cardiosphere-derived cells both genes have been shown as the most stable housekeeping genes under hypoxic conditions [15]. So far however, different housekeeping genes have not been systematically studied under hypoxic as well as differential cell density conditions together or in combination of both. In order to find the most suitable housekeeping genes for the above mentioned experimental treatments in granulosa cells, we tested seven different housekeeping genes encoding the basic transcription factor TATA binding protein (TBP), the metabolic enzymes glyceraldehyde-3-phosphate dehydrogenase (GAPDH), hypoxanthine phosphoribosyltransferase 1 (HPRT1), hydroxymethylbilane synthase (HMBS), the MHC class I cell surface molecule beta-2-microglobulin (B2M), the ribosomal proteins ribosomal protein, large, P0 (RPLP0) and ribosomal protein S18 (RPS18). The co-amplification of external standards with the respective target sequences that was routinely performed during the present study allowed the independent quantification of each of these housekeeping genes under different experimental conditions.

The conditions that we considered for current experiments include, 1) analyzing expression of the housekeeping genes in the presence or absence of FSH with respect to different cell plating density (low and high) and 2) analyzing the expression of housekeeping genes at different oxygen concentrations (normoxic vs. hypoxic) with respect to two different cell plating densities (low and high). GC were cultured under standard serum-free conditions as previously published. In earlier studies it has been shown that under these conditions bovine GC produce estrogen [16] and express the key gene of estrogen biosynthesis, *CYP19A1*[17, 18], thus suggesting their non-luteinized physiological stage. In our recent paper however, we could demonstrate that high plating density strongly reduced estrogen production and *CYP19A1* expression thus suggesting a luteinization-like physiological stage under high density conditions [10].

As housekeeping genes were reported to be regulated differentially in different tissues [19], the present work to characterize the expression of seven different housekeeping genes would be of importance for bovine ovarian somatic cell models based on cell density and hypoxia.

## Methods

### Tissue collection, follicular fluid aspiration and granulosa cell culture

Bovine ovaries were collected from a local slaughterhouse, placed and transported in phosphate buffered Saline (PBS) containing penicillin (100 IU), streptomycin (0.1 mg/ml) and amphotericin (0.5 μg/μl). Before further processing ovaries were washed in PBS with antibiotics and the health status was visually assessed. Follicular fluid along with GC were aspirated from small to medium sized antral follicles (≤ 6 mm) using sterile, non-toxic, non-pyrogenic 18 gauge needle syringes in PBS and transferred in 15 or 50 ml centrifuge tubes under sterile conditions. GC were harvested from follicular fluid by centrifugation at 500 RCF for 4 to 6 min and re-suspended in PBS. Viable cells were counted in a haemocytometer after trypan blue staining. Cells were then pelleted again and resuspended in 90% fetal calf serum and 10% DMSO (Roth, Karlsruhe, Germany) for cryopreservation. According to previous experiments the applied cryopreservation regime had no considerable effects on the physiology of thawed GC compared to freshly isolated GC as indicated by steroid production (estrogen, progesterone) and expression of marker transcripts (data not shown). For culturing cells were rapidly thawed at 37°C, washed and transferred into α-MEM containing L-Glutamin (2 mM), sodium bicarbonate (0.084%), BSA (0.1%), HEPES (20 mM), sodium selenite (4 ng/ml), transferrin (5 μg/ml), insulin (10 ng/ml), nonessential amino acids (1 mM), penicillin (100 IU) and streptomycin (0.1 mg/ml). Cells were then seeded on collagen-coated 24 well plates at two different plating densities, low density (1 × 10^5^ cells per well) and high density (1 × 10^6^ cells per well) as described previously [10]. Collagen coating was routinely implemented during this study, because according to previous experiments the number of attached and viable cells was considerably higher and no differences of marker transcript abundance levels were found between coated and uncoated plates [10]. Cells were then subjected to 7 days of basal culture (i.e. without further additives) at 37°C and 5% CO_2_. Before lysis of cells and RNA preparation cells were subjected to different treatments for 2 additional days. Experiment 1: addition of 20 ng/ml follicle stimulating hormone (FSH); Experiment 2: change to hypoxic condition (5% O_2_, 5% CO_2_, 37°C). In experimental and corresponding control samples media were changed at least every 48 h.

### Cell lysis, RNA preparation and cDNA synthesis

After nine days of incubation RNA was isolated from all samples using the Nucleo Spin® RNA II Kit (Macherey-Nagel, Düren, Germany) following the manufacturer’s instructions. Concentration of total RNA was measured three times by using a NanoDrop1000 Spectrophotometer (Thermo Scientific, Bonn, Germany). A total of 250 ng was used for cDNA synthesis using the M-MLV reverse transcriptase, RNasin ribonuclease inhibitor (both Promega), oligo-(dT) primers (2 ng/μl) mixed with random hexamer primers (4 ng/μl; both Roche, Mannheim, Germany) according to the manufacturer’s advice. cDNA was cleaned with the High Pure PCR Purification Kit (Roche) and finally eluted in 50 μl of elution buffer.

### Quantification of transcripts and of the respective external standards by real-time PCR

All primers were designed according to reference mRNA sequences by using NCBI’s pick primer software (Table 1). Quantification of transcript abundance by real time PCR was performed with SensiFastTM SYBR No-ROX (Bioline, Luckenwalde, Germany) and gene-specific primers (listed in Table 1) in a LightCycler® 96 instrument (Roche) with defined cycle conditions (Table 2) and single-point fluorescence acquisition for 10 s. Plasmids of the cloned PCR products of the respective transcripts were used as external standards. Fresh dilutions of corresponding plasmids were prepared (5 × 10^-12^ to 5 × 10^-16^ g DNA/reaction) and co-amplified during each real-time PCR run. For amplification of samples 5 ng cDNA were added to final reaction volume of 12 μl. The co-amplification of standards allowed the determination of the copy number of transcripts relative to the amount of total RNA previously subjected to cDNA synthesis.

**Table 1:** **Details of primers used for real-time RT-PCR**

Symbol	Name	Sequence
*B2M*	*Beta-2-microglobulin*	*F:ACGCTGAGTTCACTCCCAACAGCAA*
		*R:TCGATGGTGCTGCTTACAGGTCTCG*
*GAPDH*	*Glyceraldehyde-3-phosphate dehydrogenase*	*F:AGCGAGATCCTGCCAACATCAAG*
		*R:GCAGGAGGCATTGCTGACAATCT*
*HMBS*	*Hydroxymethylbilane synthase*	*F:CAGCATGAAGATGGCCCTGAAGATG*
		*R:CTCAGGTAGCAGAGGGCTGGGATGT*
*HPRT1*	*Hypoxanthine phosphoribosyltransferase 1*	*F:TGAAAAGGACCCCTCGAAGTGTTGG*
		*R:CGCCAGGTATTTCCAAACTCAACTCG*
*RPLP0*	*Ribosomal protein, large, P0*	*F:TGGTTACCCAACCGTCGCATCTGTA*
		*R:CACAAAGGCAGATGGATCAGCCAAG*
*RPS18*	*Ribosomal protein S18*	*F:GAGGTGGAACGTGTGATCACCATT*
		*R: TGTATTTCCCGTCCTTCACGTCCT*
*TBP*	*TATA box binding protein*	*F: GCCTTGTGCTTACCCACCAACAGTTC*
		*R: TGTCTTCCTGAAACCCTTCAGAATAGGG*

**Table 2:** **Real–time PCR conditions**

Step	Description	Temperature	Time
*1*	*Pre-incubation*	*95°C*	*5 min*
*2*	*Denaturation*	*95°C*	*20 sec*
*3*	*Annealing*	*60°C*	*15 sec*
*4*	*Extension*	*72°C*	*15 sec*
*5*	*Melting*	*95°C*	*5 sec*
		*70°C*	*60 sec*
		*97°C*	*1 s (continuous acquisition)*

### Statistical analysis

GraphPad prism 5.0 software was used for analysing the results with two way ANOVA. Post hoc test were performed by using quickcals post test calculator to find the significance (p < 0.05) among variables.

## Results

### Effects of FSH treatment and of different cell plating density on housekeeping gene expression

**Figure 1:**
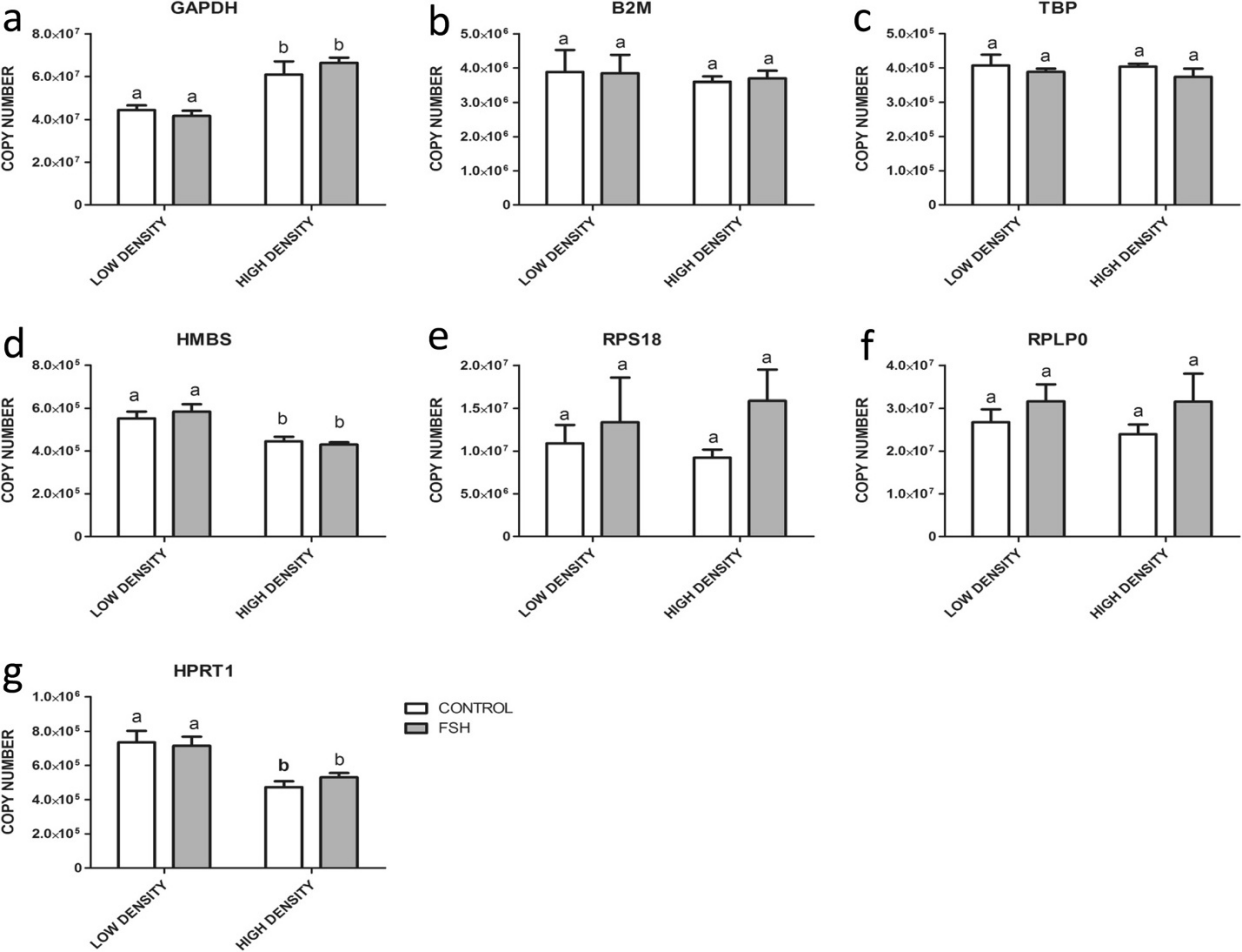
**Effects of FSH and different cell plating density on transcript abundance in cultured bovine granulosa cells.** The expression of *GAPDH* **(a)**, *B2M* **(b)**, *TBP* **(c)**, *HMBS* **(d)**, *RPS18* **(e)**, *RPLP0* **(f)** and *HPRT1* **(g)** was analyzed under FSH treatment at low and high initial cell plating density. Copy numbers were calculated and normalized to initial amounts of RNA used for cDNA preparation. Values are means ± SEMs of three independent cultures. Bars with different letters are significantly different from each other.

Three of the analyzed housekeeping genes were significantly regulated by differential plating density, with *GAPDH* showing up-, but *HMBS* and *HPRT1* down-regulation (Figure 1a,d and g). None of the genes was significantly affected by FSH treatment, however, expression of the ribosomal protein encoding genes *RPS18* and *RPLP0* seems to be marginally, but not significantly stimulated by FSH (Figure 1e and f). Only *B2M* and *TBP* (Figure 1b and c) did not show any regulation under different plating conditions or hormonal stimulation.

### Effects of hypoxia and different cell plating density on housekeeping gene expression

**Figure 2:**
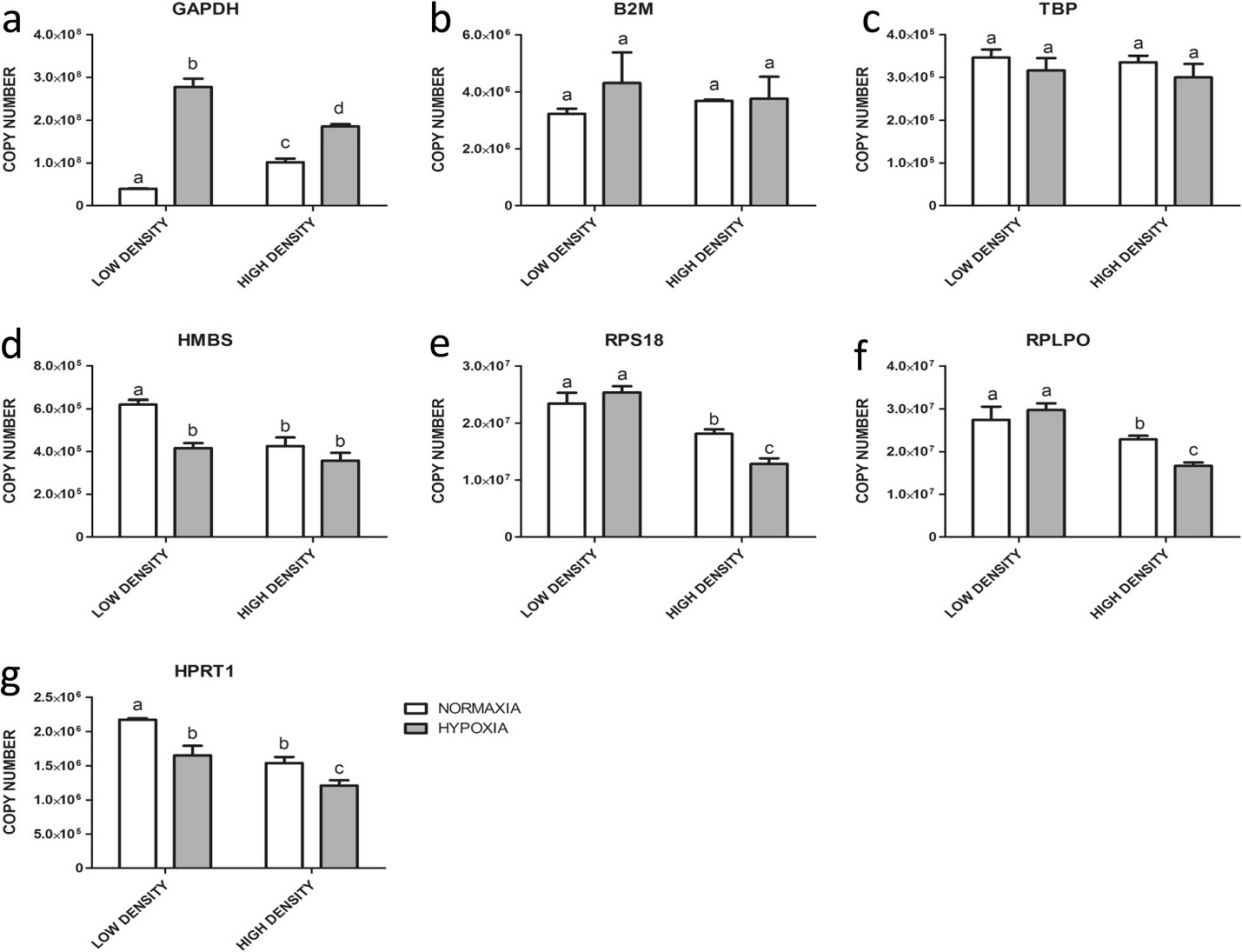
**Effects of hypoxic and normoxic conditions and of different cell plating density on transcript abundance in cultured bovine granulosa cells.** The expression of *GAPDH* **(a)**, *B2M* **(b)**, *TBP* **(c)**, *HMBS* **(d)**, *RPS18* **(e)**, *RPLP0* **(f)** and *HPRT1* **(g)** was analyzed under hypoxic (5% O_2_) and normoxic conditions (20% O_2_) at low and high cell plating density. Copy numbers were calculated and normalized to initial amounts of RNA used for cDNA preparation. Values are means ± SEMs of three independent cultures. Bars with different letters are significantly different from each other.

Under hypoxic (5% O2) compared to normoxic (20% O2) conditions, five of the genes showed significant regulation: *GAPDH* was strongly up-regulated (Figure 2a) in low and high density cultures. In contrast, the abundance of *HMBS*, *RPS18*, *RPLP0* and of *HPRT1* transcripts was lower under hypoxic conditions (Figure 2d,e,f, and g), however, in case of both ribosomal protein encoding genes, only in high (Figure 2e and f) and in case of *HMBS* only in low density cultures (Figure 2d). As for density effects and FSH stimulation, only *B2M* and in particular TBP did not show significant regulation under these experimental conditions (Figure 2b and c).

## Discussion

Appropriate normalization of real time RT-PCR data is important for transcript quantification [20]. The reliability and reproducibility of any real time RT-PCR data can be improved by including an invariant endogenous control gene (reference gene) in the assay for preventing sample to sample variations. This helps to avoid unintentionally biased data caused by varying efficiency of sample preparation such as RNA quantification, RNA integrity differences, differences in efficiency of reverse transcription and cDNA sample loading differences. Therefore, meaningful reports of any mRNA expression data require accurate and relevant normalization to some standard gene or internal control gene [21]. However, the validity of the normalization procedure largely depends on the normalizer (housekeeping gene) itself. It is of high importance to choose an appropriate control gene based on its property to maintain constant levels of expression between samples, regardless of the experimental condition. This is especially relevant when the samples have been obtained from different individuals, different tissues and/or different stages of development.

During the present study we observed significantly higher levels of *GAPDH* transcript abundance in samples plated at high compared to low density and even a massive increase of *GAPDH* expression under hypoxic conditions. These observations are in line with the view that high density of cells might create a local hypoxic microenvironment. Hypoxia can induce up-regulation of glycolysis [22] as electron transport system stops or slows down because of a lack of sufficient oxygen supply [23]. Consequently, this may lead to an increase of *GAPDH* transcripts. Therefore, the use of *GAPDH* as a housekeeping gene for normalization under direct (incubation under hypoxic conditions) or indirect (high cell density) hypoxic conditions will definitely lead to an experimental bias of relative gene expression data. Similar to *GAPDH*, also *HPRT1* and *HMBS* showed differential expression, however in the opposite direction i.e. down-regulation under hypoxic and high plating density conditions. *HPRT1* is involved in nucleotide biosynthesis. Given high cellular plating density or hypoxic conditions, the cellular proliferation decreases [10], which may lead to reduced expression of genes involved in nucleotide metabolism. The observed down-regulation of *HMBS* is also well in line with a previous study reporting that hypoxia decreases *HMBS* transcript abundance in human hepatic cell lines under hypoxic conditions [24]. Both genes encoding ribosomal proteins, *RPS18* and *RPLP0*, also do not show consistent expression under the present experimental conditions. We found down-regulation at high density and hypoxic conditions. The expressions of both genes also depends on the proliferative activity of the cells [25] which is definitely reduced at high density [10] and might be also reduced under direct hypoxic conditions. This is in line with the view of reduced protein synthesis under hypoxic and high cell density conditions and thus with reduced expression of ribosomal proteins.

In contrast to all other housekeeping genes investigated, only *TBP* and *B2M* showed a stable and constant expression irrespective of cellular plating density, oxygen partial pressure and FSH stimulation. *TBP* is an indispensable basal transcription factor which is primarily recruited to the promoter TATA box to initiate transcription by RNA polymerase-II [26]. As the basal level transcription is essential for all cellular physiological conditions, *TBP* expression might not be subjected to significant regulation. *B2M* is a cell surface marker for all nucleated cells as a component of MHC1 [27]. The expression of *B2M* usually remains constant in cultured cells unless stimulation with B-cell derived cytokines [28] to promote the synthesis of MHC1. These results seem contradictory to a previous study reporting that in cultured rat cardiosphere-derived cells *TBP* is the least, but *GAPDH* the most stable housekeeping gene under hypoxic condition. This clearly points out that the suitability of housekeeping genes for normalization has to be tested and adapted for each experimental approach.

## Conclusions

The transcript quantification with the respective external standards of different housekeeping genes revealed that *HPRT1*, *HMBS*, *RPLP0*, *RPS18*, and in particular the commonly used *GAPDH* were differentially regulated at differential cell density and different oxygen concentrations. Hence these genes are not appropriate to be used as normalizers in real time RT-PCR experiments under hypoxia. In contrast, *B2M* and *TBP* showed consistent expression levels under all experimental conditions. Therefore, we conclude that only *B2M* and *TBP* are appropriate for normalization in bovine ovarian granulosa cells under FSH stimulation, differential oxygen concentrations and varying cell density.

## Abbreviations

*ACTB*: β-actin
*B2M*: MHC class I cell surface molecule beta-2-microglobulin
DMSO: Dimethylsulfoxide
FSH: Follicle stimulating hormone
*GAPDH*: Glyceraldehyde-3-phosphate dehydrogenase
GC: Granulosa cells
*HMBS*: Hydroxymethylbilane synthase
*HPRT1*: Hypoxanthine phosphoribosyltransferase 1
IGF-I: Insulin-like growth factor 1
LH: Luteinizing hormone
MEM: Minimum essential media
PBS: Phosphate buffered saline
*RPLP0*: Ribosomal protein, large, P0
*RPS18*: Ribosomal protein S18
RT-PCR: Reverse transcripton - polymerase chain reaction
*TBP*: TATA binding protein.
